# The Actions of Lithium on Glaucoma and Other Senile Neurodegenerative Diseases Through GSK-3 Inhibition: A Narrative Review

**DOI:** 10.7759/cureus.28265

**Published:** 2022-08-22

**Authors:** Arihant Singh, Tanishq Kumar, Vivek R Velagala, Swedaj Thakre, Abhishek Joshi

**Affiliations:** 1 Medicine, Jawaharlal Nehru Medical College, Datta Meghe Institute of Medical Sciences, Wardha, IND; 2 Community Medicine, Jawaharlal Nehru Medical College, Datta Meghe Institute of Medical Sciences, Wardha, IND

**Keywords:** β-catenin pathway, senile, glaucoma, lithium, dementia

## Abstract

Glaucoma can be described as a set of progressive optic neuropathies. They cause a gradual, irreversible loss of the field of view, which concludes in complete blindness. Evidence suggests that patients who have glaucoma face a greater risk of suffering from senile dementia. Dementia is a group of conditions that occur in old age individuals. Neurodegeneration is a characteristic pathological feature of dementia, the progression of which causes a decline in cognition, which may be accompanied by memory loss. Severe dementia in old individuals usually presents as Alzheimer’s disease, which significantly contributes to a load of dementia in India. Parkinsonism is another common neurodegenerative disease that is known to occur in the elderly.

The WNT (Wingless-related integration site)/β-catenin pathway is a multistep process that is responsible for the regulation of various cellular functions. Lithium can up-regulate this pathway by disrupting Glycogen synthase kinase-3β (GSK-3β). This action of Lithium can effectively counteract neuroinflammation and neurodegeneration. The current use of Lithium remains majorly confined to its use for episodes of mania in bipolar disorder (BD). However, recent literature gives insight into how Lithium can improve the visual field in glaucomatous eyes. Symptomatic improvement after lithium administration is seen as it has neuroprotective actions on the retinal ganglion cells (RGCs). Prolonged lithium use improves axonal regeneration and neuronal survival. Lithium also improves the worsening of symptoms in other dementia-related neurodegenerative diseases like Alzheimer’s and Parkinsonism. The physiological actions of Lithium can be utilized in providing effective, holistic therapy options in pathologically related senile degenerative disorders. Significantly better results can be obtained if Lithium therapy is given in conjunction with the drugs used to manage these disorders.

## Introduction and background

Glaucoma is a neurodegenerative disease that involves a group of eye-related neuropathies with a progressive nature and is caused due to slow destruction of retinal ganglion cells (RGCs). Glaucoma affects the retinal deposition of Beta-amyloid, phosphorylated Tau protein (pTau), and synuclein. The condition causes the reduction of visual field and functional impairment in the patient, which is attributed to the progressive nature of axonal degeneration in the retina [1]. Glaucoma is widely prevalent and contributes significantly to the global burden of blindness. It poses a great threat to global public health, as 111.8 million people are estimated to suffer by 2040 [2,3]. Most cases of glaucoma which involve the global population are that of primary angle-closure glaucoma (PACG), which occurs because of obstruction of the narrow anterior chamber angle. Another common type of glaucoma is simple chronic glaucoma, which is also known as primary open-angle glaucoma (POAG) [4].

It has been shown in many studies that patients who are diagnosed with glaucoma involve a higher risk of developing senile dementia [5]. Both primary open-angle glaucoma and old-age dementia are diseases that are known to be more common in the elderly. Neurodegenerative diseases can affect both the retina and the brain. They include glaucoma, Alzheimer’s disease (AD), and Parkinson’s disease (PD) [6].

Dementia can be described as a type of neurodegenerative disorder with the characteristic features of a steep cognitive decline, progressive loss of memory, and changes in behavior. It usually occurs in people who belong to the senile age group. Dementia severely hampers the patient’s ability to perform day-to-day tasks, affecting their functionality. The most common form of dementia that affects senile populations is Alzheimer’s disease. It involves the senile degeneration of the cerebral cortex, which results in the loss of proper cognition and loss of memory. Parkinson’s disease is described as a locomotor condition involving gradual neurodegeneration of the dopaminergic neurons in the brain. Degeneration mainly occurs in the substantia nigra.

Immunocompetent, neuroprotective cells of the central nervous system (CNS) are known as Microglia. The reaction of microglia to injury involves changes in morphology, including inflammatory cytokine production and proliferation. If the microglial response due to sustained CNS damage is left unchecked, the rates of neuronal survival fall [7]. The etiology of all senile CNS disorders includes a neuroinflammatory response, in which the microglial cells play a crucial role.

The WNT (Wingless-related integration site)/β-catenin pathway is a mechanism involved in various processes that maintain homeostasis. These include the proliferation of cells, organogenesis, embryogenesis, and apoptosis. This pathway can be affected and may present as psychiatric, neurological, and cancerous changes. WNT/β-catenin pathway significantly impacts the pathological progression of diseases with neuronal degeneration, like glaucoma. Glycogen synthase kinase-3β (GSK-3β) is a vital controller which can also influence the cascade [8,9]. It acts negatively on the cascade as it is an inhibitor. Inflammation occurs following a diminution in WNT/β-catenin pathway mechanisms. GSK-3β upregulation also causes an inflammatory response. Therefore, drugs or processes which inhibit GSK-3β help in neuroprotection.

Lithium is a drug used extensively to treat recurrent mania episode cycles in bipolar disorder (BD) cases [10]. It can mediate its actions through many intracellular cascades, which include GSK-3β. Its use in patients with glaucoma, Parkinson’s, and Alzheimer’s disease has not been studied extensively. However, due to its positive action on the WNT/β-catenin pathway through GSK-3β inhibition, it should be possible to elicit its protective effects.

Glaucoma, Alzheimer’s, and Parkinson’s disease have overlapping etiological, pathological, and pharmaceutical domains. AD and PD are age-related disorders and are seen in the senile population. Glaucoma has an increased prevalence rate after the age of 40 years [11]. All these diseases are governed by the WNT/β-catenin pathway. Regulators for this pathway could significantly control these diseases.

The review is undertaken to effectively assess the actions of Lithium therapy on glaucoma and other neurodegenerative diseases, which commonly affect the senile population. Their pathophysiology and correlation with the WNT/β-catenin pathway have been reviewed, which may help in the development of effective therapies in the future.

## Review

Glaucoma

Glaucoma is a progressive ophthalmological condition of neurodegenerative nature. It is also responsible for a large percentage of irreversible blindness worldwide [12]. It presents clinically with a retinal ganglion cell (RGCs) decline, accompanied by the thinning of the retinal nerve fibers. Glaucoma is characterized by optic disc cupping. Glaucoma risk factors include increased intraocular pressure, aging, and genetic predisposition [4].

Epidemiology, etiology, and therapy of glaucoma 

Glaucoma contributes to the second largest patient load as an ophthalmic condition that leads to blindness. It is topped only by cataracts, making it an alarming issue for global health. The loss of vision caused by glaucoma is usually irreversible due to neurodegeneration. It was estimated that primary open-angle glaucoma (POAG) affects about 57.5 million people worldwide [13]. Over 11.2 million Indians over the age of 40 years showed glaucomatous changes associated with adult-onset glaucoma. Primary open-angle glaucoma affects approximately 6.48 million people, whereas 2.54 million cases of primary angle-closure glaucoma are estimated in India alone [14]. POAG may be predisposed in individuals with an elevation in Intraocular Pressure (IOP), genetic factors, age, diabetes, hypertension, and cigarette smoking. Age, female gender, and hypermetropic eyes are the main etiological factors that may lead to the formation of PACG. The prevalence of simple chronic glaucoma is known to rise with age and usually affects people in the 6^th^ and 7^th^ decades of life. Age is an established prevalence factor for open-angle glaucoma, making glaucoma one of the many reasons for irreversible loss of vision in the aging population [15]. Other factors affecting the progression or onset of glaucoma include chronic use of steroids and a corneal thickness of less than 5 mm. Latanoprost, travoprost, timolol, and betaxolol are drugs commonly used in the treatment of POAG. Drugs used for PACG include timolol, betaxolol, and latanoprost. New medications, which include latanoprostene bunod and netarsudil, are effective treatment options [16]. The incidence of POAG and PACG in India is shown in Table 1.

**Table 1:** Incidence of POAG and PACG in India

	Primary Open-Angle Glaucoma	Primary Angle-Closure Glaucoma
Number of cases in India over 40 years of age (In millions)	6.48	2.54

Pathophysiology of glaucoma

The death of Retinal Ganglion cells is directly related to the intraocular pressure of the eye, which is controlled by the equilibrium between the uveoscleral pathway and the trabecular meshwork. Pathophysiology of glaucoma is based on the rise in intraocular pressure, which may be due to elevated resistance to the aqueous humor outflow from the trabecular meshwork or an obstruction in the drainage pathway.

Mechanical straining occurs in the lamina cribrosa of the eye due to excessive intraocular pressure [17]. The optic nerve fibers exit the eye in the region of the lamina cribrosa, where the sclera is perforated. This makes the lamina the structurally weakest section of the posterior eye wall. This straining of the lamina cribrosa can cause its remodeling, followed by axonal damage and disruption of effective neuronal transport [18,19]. Axonal transport disturbance results in the accumulation of vesicles. It also results in pre-laminar and post-laminar disorganization of neurofilaments and microtubules. Glaucoma can also occur in eyes with normal values of intraocular pressure, which may be caused due to a high-pressure gradient across the lamina cribrosa. Day-to-day activities of old patients are affected due to a significant loss of peripheral vision, which can dramatically elevate the incidence of bone fractures due to accidental falls in the population. Apart from optic neuropathy, glaucoma is associated with abnormalities in color perception and contrast of vision [20].

WNT/ β-catenin pathway

**Figure 1:**
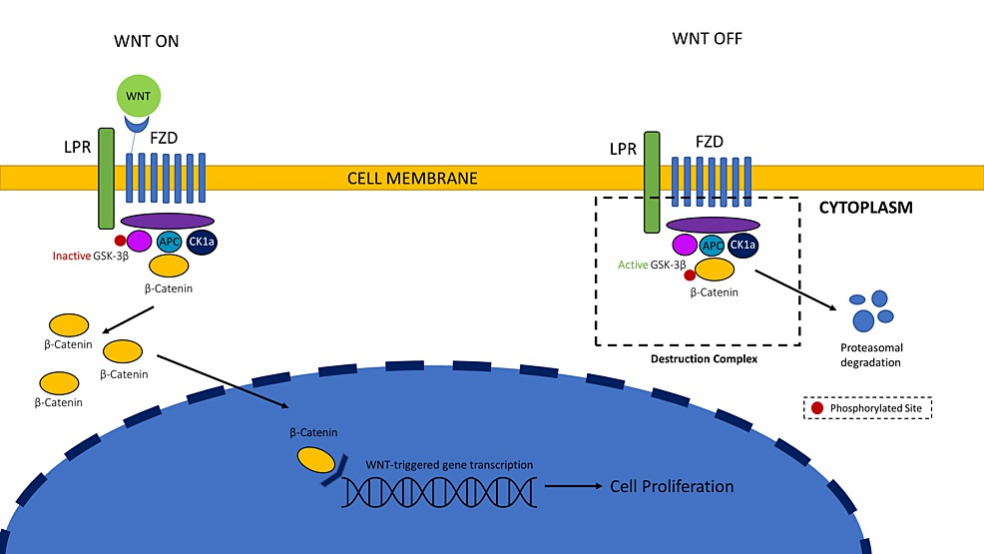
WNT/ β-Catenin Pathway: "WNT ON": Binding of the WNT proteins to the FZD and the LPR co-receptor decreases the activity of GSK-3β. This allows for increased cell proliferation. "WNT OFF": When no ligand is present, the phosphorylation of β-catenin takes place. This causes the degradation of proteins. FZD: Frizzled receptor, GSK-3β: Glycogen synthase kinase-3β The image is created by the authors.

The WNT/β-catenin pathway is a molecular cascade that regulates the fate of a cell. It has many regulators, inhibitors, and activators. One of the major inhibitors of this cascade is Glycogen synthase kinase-3β (GSK-3β). It is an essential factor in many cellular signaling pathways. GSK-3 has two different isoforms, α, and β. GSK-3β has been found to influence the control of various pathophysiological pathways, including inflammation and cell polarity. The downregulation of the WNT/β-catenin pathway and upregulation of GSK-3β is directly related to inflammation, which is an age-related mechanism [21]. Figure 1 shows the functioning of this pathway.

It has been established in recent studies that the WNT/β-catenin pathway is majorly responsible for the progression of glaucomatous eyes [21]. The cascade directly affects the cells of the Trabecular Meshwork (TM) of the eye. It was also believed that this pathway could help in the management of intraocular pressure (IOP) [22]. The WNT/β-catenin pathway can be exploited to derive a fresh treatment strategy that targets patients with glaucoma and other associated conditions. Lithium chloride can activate the WNT/β-catenin pathway of the Trabecular meshwork cells to diminish the production of matricellular proteins [23]. Any down-regulation in the WNT/β-catenin pathway and K-cadherin can elevate IOP in glaucomatous eyes [24]. The number of studies describing Lithium’s action on glaucoma is extremely limited. The actions of Lithium are seen in patients after a long duration of therapy, with consistent dose monitoring.

Alzheimer’s disease

A neurological disease with the pathognomonic extracellular accumulation of Beta-amyloid plaques and intracellular accumulation of neurofibrillary tangles in the brain is called Alzheimer’s disease. Hallmarks of the disease also include the collection of hyperphosphorylated tau protein (pTau) in the intraneuronal segment of the optic nerve and the retina. The usual onset is insidious, and the progression is slow. The drugs usually used for AD include Galantamine, donepezil, and rivastigmine. In Alzheimer’s patients, death of neurons and general pathogenesis of patients is associated with varying levels of GSK-3 [25]. Research demonstrated that Lithium not only reduces phosphorylated tau by the inhibition of GSK-3 but also prevents its hyper-phosphorylation [26,27]. The continuation of long-term lithium treatment can significantly reduce Alzheimer’s-associated tau-phosphorylation and degeneration of axons.

Parkinson’s disease

Parkinson’s disease or parkinsonism is a neurological condition that affects the basal ganglia and is associated with the common symptoms of resting tremors, muscular rigidity, and unstable posture. PD can also be associated with dysarthria, constipation, and dysphonia. Histologically, Parkinson’s disease shows Lewy body inclusions with aggregates of α-synuclein. The disease also involves the degeneration of retinal dopaminergic cells.

The pathophysiology of Parkinson’s disease includes the combination of endoplasmic reticulum (ER) stress and defective protein degradation, like other diseases which involve the degeneration of neurons. A vast number of studies have been performed on the actions of Lithium in old-age Parkinson’s disease patients. Lithium prevents cells from accumulating lipids stimulated by ER stress as a consequence of GSK-3β inhibition [28]. Administration of chronic low-dose Lithium has shown neuroprotective properties, probably due to the regulation of the stress gene expression [29]. In a study, 1-methyl-4-phenyl-1,2,3,6-tetrahydropyridine (MPTP) lesioned mice were administered low-dose combinations of Lithium along with L-dopa and sodium valproate. It was found that these combinations helped reduce the neurotoxin-induced involuntary movements and elevated the density of neurons in the brain, mainly dopaminergic fibers in the substantia nigra [30]. Observations indicated that the concentrations of dopamine metabolite, dihydroxyphenyl acetic acid (DOPAC), were reduced after the administration of these lithium combinations.

Studies performed in animal models of Parkinson’s disease show that the action of Lithium can prevent dopamine depletion. It stimulates an over-expression of B-cell lymphoma 2 protein (Bcl-2) and downregulation of bcl-2-like protein 4 (Bax), which is responsible for its anti-apoptotic effect [31]. The up-regulation of Bcl-2 is accountable for providing neuroprotection against dopaminergic neurotoxins.

Lithium

Lithium chloride is a psychiatric medication that was introduced in 1949. Since then, it has been used as psychotherapy for many chronic mental disorders. It is a popular treatment choice in bipolar disorder patients and is also used in the management of traumatic brain injury [8]. Lithium is a useful drug in diseases associated with neurodegeneration, which include Parkinson’s, Huntington’s, and Alzheimer’s disease [32]. Lithium usage has also shown a relative fall in suicidality in patients suffering from these disorders [33].

Even though Lithium was a popular drug for treating bipolar disorder, the number of lithium prescriptions started decreasing due to rising concerns about lithium toxicity. Lithium therapy, if not regulated by therapeutic monitoring, can include side effects like excessive thirst, tremors, hypercalcemia, and weight gain. Periodic assessment through blood tests is important as lithium can affect the functioning of the parathyroid and thyroid glands [34].

The use of Lithium also declined due to the increase in the marketing of alternative treatments and medications [35]. The use of Lithium was greatly affected by the introduction of sodium valproate. Due to outdated evidence-based studies, prescription rates also decreased due to increasing suspicion about Lithium’s efficacy.

Studies with updated and more reliable designs were done on Lithium. These included meta-analyses and double-blind, randomized controlled trials, which report Lithium as an effective drug for the therapy of BD, as it is helpful in the prophylaxis of new manic and depressive episodes in the long term [36]. Combination therapy of Lithium and first-generation antipsychotics was a popular choice for the treatment of BD. First-generation antipsychotics were replaced eventually by atypical antipsychotics due to cardiac side effects of the former [37].

Lithium is not only beneficial in mood disorder therapy but also in some neurodegenerative syndromes. This is due to the neuroprotective effect of Lithium, which guards neurons against excitotoxic and ischemic damage [38].

**Figure 2:**
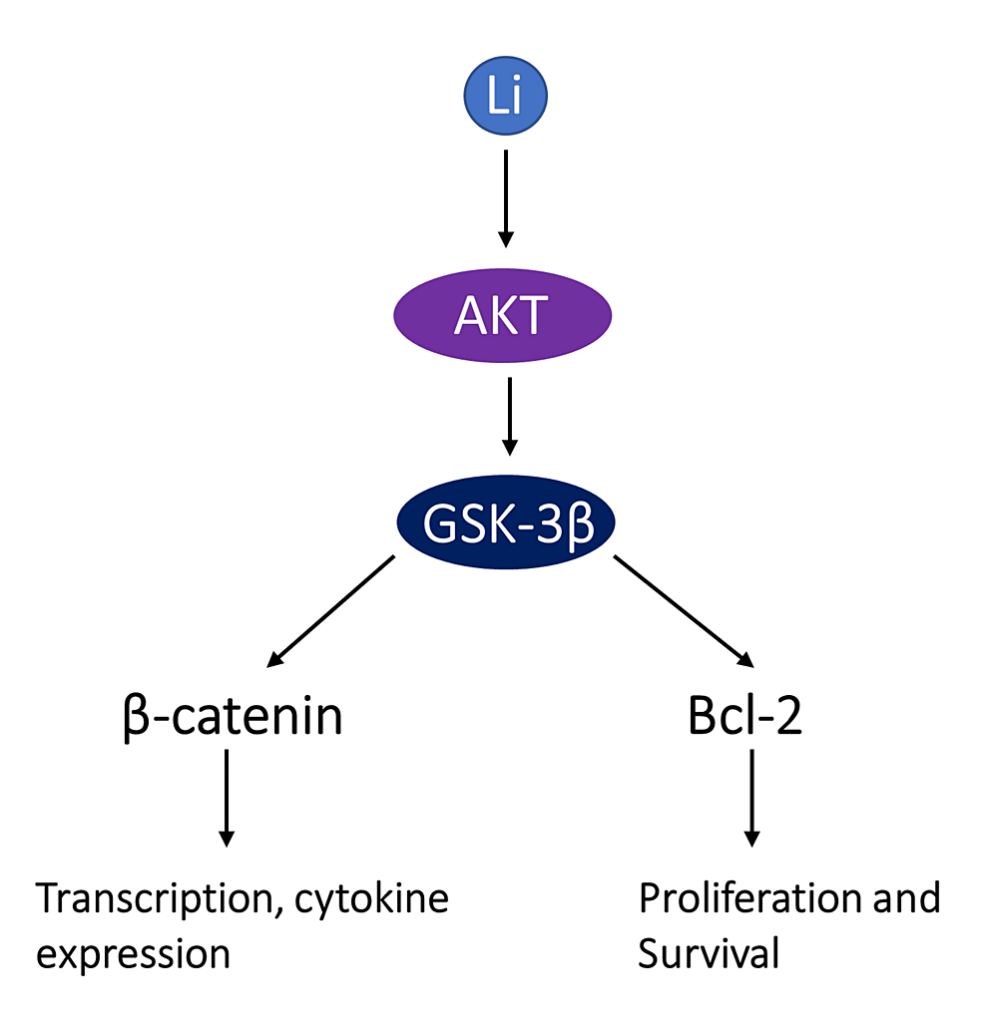
Action of Lithium on the PI3K/Akt pathway GSK-3β; Glycogen synthase kinase-3β, Li: Lithium The image is created by the authors.

Lithium exerts its actions by acting on Retinal Ganglion Cells (RGCs). It is found to increase the chances of neuronal survival and axon regeneration significantly in animal models [39]. Therapeutic actions relevant to neuroprotection are seen best at the recommended concentrations for therapy of bipolar (0.5-1.2 mM). Significant results are seen after long-term therapy [40]. Lithium’s actions can be relayed through various signaling mechanisms, mainly GSK-3β. Neuronal survival and regeneration of Retinal ganglion axons are supported by Lithium through the Bcl-2-dependent mechanism [41]. Bcl-2 is a family of proteins that modulate apoptosis. They were first discovered in 1984 and were found to inhibit cell death. The mammalian Bcl-2 family of proteins consists of about 30 related proteins. Bcl-2 is an essential regulator for the control of both nerve survival and axon regeneration [42]. Lithium can stimulate the phosphatidylinositol-3-kinase/protein-kinase-B pathway (PI3K/Akt pathway) to up-regulate Bcl-2 expression [43]. Lithium can help in neuroprotection through the inhibition of the N-methyl-D-aspartate (NMDA) receptor and glutamate-induced AKT activity [44]. Figure 2 shows the action of Lithium on the PI3K/Akt pathway [23].

Neuro-protective properties of Lithium suggest that its use may help in the treatment of diseases involving nerve damage and degeneration of RGCs, like glaucoma, optic-nerve neuritis and other spinal cord and brain disorders [40]. For effective regeneration of neuronal damage, it is crucial to employ lithium therapy with other drugs that create favourable conditions for the induction of suitable conditions for the drug to act effectively.

## Conclusions

After reviewing the available literature, significant pathophysiological connections were found between Alzheimer’s, Parkinson’s disease, and glaucoma, which can be exploited to provide effective treatment options. The use of lithium therapy has seen a significant blow due to the ascent of alternatives and the devastating effects of its toxicity. However, Lithium use in old age patients over a long treatment period can not only delay neurological cell death but also prevent the destruction of RGCs. The study of lithium-like drugs, which up-regulate the WNT/β-catenin pathway, should be encouraged as this pathway can be an important new target for the effective management of glaucomatous symptoms. Suitable clinical research trials must follow the pharmaceutical effects of such drugs.

When given under therapeutic monitoring and dose moderation for a prolonged period, it can have a plethora of positive effects ranging from the reduction of patient suicidality to diminished mitochondrial fission. Studies have also described Lithium as an effective drug for the management of acute mania episodes, which was its initial intended use. Even though new drugs like valproate offer lesser side effects, Lithium provides a better guard against recurrent episodes. It also helps keep associated neuronal degeneration under control, providing a holistic approach to the treatment of these interconnected diseases.
